# Workplace diversity in female-dominated industries and intraindividual identity conflict

**DOI:** 10.1371/journal.pone.0321040

**Published:** 2025-05-06

**Authors:** Magdalena Calderón-Orellana, Raúl Berríos

**Affiliations:** 1 Escuela de Trabajo Social. Pontificia Universidad Católica de Chile, Santiago, Chile; 2 Departamento de Trabajo Social. Universidad de Concepción, Concepción, Chile; 3 Departamento de Administración, Facultad de Administración y Economía, Universidad de Santiago de Chile, Santiago, Chile; Harvard Medical School, UNITED STATES OF AMERICA

## Abstract

This research paper examines the impact of gender and socioeconomic status (SES) diversity on intraindividual identity conflict within a female-dominated economic sector. The study also evaluates whether this relationship is moderated by gender and SES, as suggested in prior research. A quasi-experimental design was employed with 186 child protection workers as participants. Using vignettes that depicted varying levels of diversity, participants were randomly assigned to different conditions. The degree of dissimilarity was determined by comparing the diversity level described in the vignette with the participants’ individual characteristics, and intraindividual identity conflict was subsequently measured. Findings revealed that SES dissimilarity within a workgroup is associated with heightened intraindividual identity conflict. Furthermore, the study confirmed that SES moderates the relationship between diversity and intraindividual identity conflict. This research contributes to the existing body of knowledge on the effects of diversity on group identity conflicts, with a particular focus on intraindividual conflict in feminized occupations. Additionally, the findings on SES diversity underscore the importance of advancing our understanding of socioeconomic or class diversity and expanding the study of diversity beyond the Global North—topics that have been largely overlooked in the literature.

## Introduction

The study of workplace diversity has shown an upsurge of interest, despite persistent challenges in interpreting this complex phenomenon [1–3]. One common finding in literature is that greater gender diversity at work, as well as other sociodemographic identities, such race or nationality, tend to yield adverse outcomes for individuals and work groups. This is primarily due to the inherent group conflicts engender multiple identities in diverse contexts [4,5]. The negative effects of diversity can be explained through the lens of social categorization, which posits that visible demographic differences prompt individuals to compare and distinguish themselves from others. This differentiation leads identity groups to compete for relative status, thereby generating conflict [6–8].

However, limited attention has been given to understanding the intraindividual dynamics stemming from the interplay between diversity and identity conflict. Intraindividual identity conflict refers to the inconsistency between values, beliefs, norms, and demands inherent in two or more identities [9]. Individuals, influenced by the demographic makeup, group diversity, and the processes of categorization and self-categorization, may adopt or reject certain social identities based on their perceived benefits or detriments [10]. These shifts in the valuation and acceptance of individual identities can create tensions, leading to conflict.

Previous research linking diversity and identity conflict has mainly focused on the perception of identity conflict and the conflict between just two identities, often occupation and gender, without considering other social identities [11]. Such analyses limit our understanding of identity conflict since the conflict between two identities can impact the boundaries with other individual identities [12]. For instance, a woman working in a male-dominated occupation may experience identity conflict between her gender and occupation. In response to this conflict, she may strengthen her organizational identity or another valued identity in that work context.

Noticeably, the accrued research has focused on masculinized occupations, which raises questions about intraindividual identity conflicts in feminized occupations, such as the care sector examined in this research. This seems an important aspect of research considering the longstanding evidence linking conflict of women in current society [13–15]. Examining the relationship between diversity and intraindividual identity conflict within a highly feminized industry offers an opportunity to gain insights into the patterns observed in other studies exploring the detrimental effects of diversity, especially considering that the outcomes of diversity are contingent on the status of identity [2].

Nonetheless, most research investigating diversity and identity conflict has focused on prominent social features that characterize dilemmas in American society, such as race, often neglecting other pervasive features found in many societies worldwide, such as class or socioeconomic status. In this context, incorporating the socioeconomic dimension into the study of diversity is crucial, as it is one of the many relevant identities that intersect and explain the exclusion experienced by workers. It acts as a barrier to their full participation in both the workplace and society as a whole [16].

This study incorporates SES diversity as a significant social determinant and assesses intraindividual conflict as a confluence of multiple identities activated and processed by individuals. This underscores the idea that a comprehensive understanding of gender diversity must encompass other identities to better grasp the gender experience [17].

Expanding our understanding of the relationship between diversity and intraindividual identity conflict is important because of the implications of conflict for several work-related outcomes. Intraindividual identity conflict has been negatively associated with well-being, motivation, job satisfaction, and performance. For example, Settles [18] notes that identity conflict between female and scientific identities is associated with lower levels of performance and well-being. Similarly, the activation of conflicting identities is related to a decrease in well-being and self-esteem among those who consider themselves stable in their identity [19]. Ramarajan, Rothbard and Wilk [20] observed the negative relationship between identity conflict and the performance of tasks that involve interactions between people. Finally, Gibson, Dunlop, and Raghav [21] empirically examined identity conflict in workers of a multinational company, confirming that the less intraindividual identity conflict, the more the workers thrived at work.

Thus, this study aims to understand the effect of gender and SES diversity on intraindividual identity conflict and to examine how socioeconomic status and gender moderate this hypothesized association in a female-dominated sector. Therefore, our study sheds light on a reality that has traditionally been overlooked in diversity research: feminized sectors and occupations, particularly in the Global South, specifically South America, and low-level positions [22]. In summary, this study centers on women engaged in “dirty jobs” that cater to stigmatized populations [23] and are often perceived as having lower societal value [24].

In doing so, we contribute to the theory by *(a)* Adding counterfactual evidence from a sample of female-dominated occupations (child protection workers), which permits us to set the boundaries for advancing new hypotheses in this research domain. *(b)* reversing the lens of the well-studied relationship between diversity and conflict, focusing now on the intraindividual consequences of diversity rather than the interpersonal domain. *(c)* Considering a new focus of attention in the study of diversity, moving from traditional studies that describe the realities of the global north, into another pervasive socioeconomic determinant (i.e., socioeconomic status). *(d)* Observing a reality that has been underreported until now, namely the reality of Latin American countries, highlighting identities that differ from those constructed in the Global North. While diversity may be a constant in the workforce, the components upon which the phenomenon is built will vary depending on culture [25].

### Diversity and intraindividual identity conflict

Organizational diversity is a double-edged sword, as it involves benefits and limitations for individuals, groups, and organizations [26–28]. Groups that are more diverse regarding formation or occupation are more creative and innovative. At the same time, non-work-related demographic diversity, such as race and gender, it often leads to negative outcomes like decreased group cohesion, increased conflict, and reduced performance [4,29–32].

This double-edged characteristic related to diversity can be understood through the lens of social identity theory [33]. According to this theory, individuals naturally seek to belong to groups they value, activating their social identities. Consequently, through cognitive and emotional evaluation, people aim to be part of groups that benefit them while distinguishing themselves from groups they perceive as detrimental to their self-concept. However, striking this balance often generates intergroup tensions [10].

This dynamic process also operates at the individual level. When a person perceives a significant difference between themselves and their group and considers this difference personally relevant, they tend to categorize their environment into an “us” versus “them” framework [6]. As identity boundaries are fluid and adaptable [34,35], individuals who deviate from their group seek to develop or limit the identity that sets them apart. Modifying this differentiating identity can have consequences for the boundaries of other identities [20]. Consequently, individuals may experience conflict between their identities as they balance the maintenance or abandonment of meanings, values, and behaviors associated with one identity to preserve another [20].

Consider gender as an example. An individual who differs from their work group in terms of gender and values their gender may choose to emphasize or deemphasize their identification with that gender identity. These adjustments can generate tensions and conflicts with other identities, such as occupation or seniority, as individuals strive to establish or maintain an identity that aligns with their self-concept [12].

The evidence regarding diversity and intraindividual identity conflict is scarce, although needed [3]. In this line, the most representative published work is the one developed by Veldman and her colleagues [11], who studied the effect of gender relational diversity, understood as dissimilarity, on the perception of identity conflict between gender and occupation. The study, conducted in a highly masculinized industry, found that gender dissimilarity was related to a perceived identity conflict for women but not for men. Also confirmed that identity conflict was related to identification with the team and turnover intentions, symptoms of burnout, lower job satisfaction, lower work motivation, and lower perceived performance.

Based on this previous research and theory, it is expected that individual differences based on demographic characteristics, such as gender or SES, will surface as the identity that distinguishes a person from the group. Therefore, a prominent characteristic implies that multiple identities’ boundaries are adjusted and mobilized simultaneously, causing intraindividual conflict. Thus, we advance the following hypotheses.

*Hypothesis 1* Gender diversity, understood as dissimilarity, has a positive, and significant relationship with the intraindividual identity conflict of child protection workers.

*Hypothesis 2* SES diversity, understood as dissimilarity, has a positive, and significant relationship with the intraindividual identity conflict of child protection workers.

Previous studies have established that being a minority or otherwise different from a group concerning demographic characteristics can negatively affect individuals. Simultaneously, it has been posited that context and status affect the result of group diversity [2,36,37]. Consequently, the impact of dissimilarity within a group is contingent upon the status of individuals [38].

Similarly, Veldman et al. [11] establish that being distinct from the group in a male-dominated occupation negatively affects intraindividual identity conflict, particularly among women. As such, it is plausible to surmise that gender dissimilarity within highly feminized occupations, such as child protection workers, may have a more pronounced impact on men. However, given the historical observation of male privilege in this field [39,40], we propose that the effect of gender will differ from what is observed in a male-dominated occupation. Indeed, in social and care services, masculinity is associated with power and status [41].

Thus, based on evidence associated with the value of status in demographic diversity outcomes [36,37], we consider that gender will condition the hypothesized effect of group diversity on identity conflict, affecting women more. Conversely, regarding SES diversity, we believe that people of lower socioeconomic levels will be more affected since they live a double exclusion due to being different from their team and having a lower status [42]. In this way, we propose that:

*Hypothesis 3* The effect of gender diversity, understood as dissimilarity, on intraindividual identity conflict in child protection workers will depend on gender, being most pronounced in women.

*Hypothesis 4* The effect of SES diversity, understood as dissimilarity, on intraindividual identity conflict among child protection workers will depend on SES, being most pronounced at lower socioeconomic levels.

**Fig 1:**
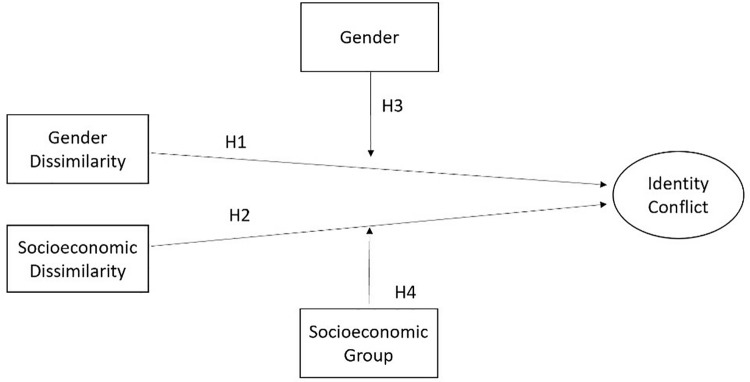
Research theoretical model.

Fig 1 provides a visual representation of the theoretical model.

## Materials and methods

The model was evaluated through a quasi-experimental research design employing vignettes. Vignettes are a method that introduces the independent variable – dissimilarity in this case- into specific scenarios presented to the subjects under study [43]. Specifically, the research design considered the random assignment of vignettes, and consequently, the specific condition of the independent variable, which allowed for the integration of internal validity, a characteristic commonly associated with experimental designs, as well as external validity, a hallmark of non-experimental designs [44].

In addition to reasons related to the validity of the study, a quasi-experimental design was employed to complement existing findings on diversity, which have traditionally been derived from observational research [45,46].

### Population and sample

The population corresponds to child-protection workers dependent of NGOs in Chile. Contact was made through a union of subcontracted workers of the Childhood Protection Service of the Ministry of Social Development and Family. The initial sample considered four hundred people contacted through email sent by the union’s leadership, and 186 responses were obtained, corresponding to 46.5% of the total sample. The description of the sample is presented in Table 1.

**Table 1:** Sample Characteristics.

Variables	n	%	Min	Max	Mean	Median	Standard deviation
**Gender**							
**Men**	27	14.5					
**Women**	158	84.9					
**Non-binary person**	1	0.5					
**Age (years)**			23	60	33.65	32.65	7.20
**Tenure (years)**			0.5	18	3.59	3	2.95
**Objective SES**	186						
** 1 (poor people)**	0	0					
** 2 (vulnerable people)**	12	6.7					
** 3 (lower middle class)**	30	16.7					
** 4 (typical middle class)**	50	27.8					
** 5 (emerging middle class)**	46	25.6					
** 6 (affluent middle class)**	40	22.2					
** 7 (high class)**	2	1.1					
**Missing System**	6						

The majority of participants were women (84.9%), relatively young (average age of 33 years), and highly educated (96.8% declared that they had completed higher education, and more than a third of the sample enrolled in postgraduate studies). The sample also showed instability and turnover when considering the average tenure, which reached 3.59 years, while 75% of the sample had worked fewer than five years. Based on the participants’ socioeconomic characteristics and AIM [47] methodology, it is possible to speculate that the majority are part of the middle class. Specifically, more than 50% is defined as middle and emerging middle classes. These results are consistent with studies conducted for populations working in social-care services for children in Chile and other contexts, such as China, the USA, and different countries in Europe [48–51].

### Scenarios and vignettes

Vignettes were used to manipulate the independent variable, introducing varying levels of gender and SES diversity to participants. The information in the overall scenario remained consistent for all participants, while the information defining the level of diversity of the respondent’s supposed working group was altered, as detailed in the following vignette example:

*Imagine that in your workplace, after an analysis of employees’ characteristics, it has been determined that the majority are women, and most workers belong to high socioeconomic status. Therefore, to increase diversity and ensure that the organization is made up of workers of different characteristics, hiring people who meet the requirements, have merits and are mainly men and people of medium and low socioeconomic levels is promoted.*

*After six months of implementing this practice, its results can already be observed; in this case, the workgroup has become more diverse. This means that the workgroup is now composed of equal numbers of women and men and an equal number of low and high-socioeconomic-status workers.*

Each vignette provided a scenario in which gender and SES diversity levels were combined. Six vignettes were created and randomly assigned using the randomization function within the Qualtrics© survey software.

The level of diversity was described in the last paragraph of the vignette. Gender diversity group and the SES diversity group were conceived as dissimilarity based on the definitions put forth by Harrison and Klein [1]. Generically, dissimilarity is the difference between an individual and their coworkers regarding a specific characteristic [52].

The vignette manipulation check was conducted using an ANOVA test, including the means between the six groups that responded to the six vignettes. The ANOVA results indicated that the vignettes do not relate to identity conflict (*F* [5], 180) = 0.46; *p* = 0.81), preventing problems associated with common method variance bias.

## Measures

### Gender and SES dissimilarity

Based on the vignette, the gender self-reported identity and categorical SES, the variables gender dissimilarity and SES dissimilarity were constructed by the researchers as an ordinal variable assessing the relationship between the individual and the group at three levels: low (when the person, concerning the group, represented the majority), medium (when the person was equally represented about the group), and high (when the person, about the final group, constituted a minority). This computation correspondingly resulted in a numerical value ranging from 1 to 3.

For example, if a woman was presented with a vignette describing the final group primarily composed of women, her level of gender dissimilarity was low. Whereas if a man faced the same vignette, his level of gender dissimilarity was high. In the case of SES dissimilarity, if the vignette presented a final group composition with a high-class people majority and the respondent was a high SES person, the dissimilarity was low. On the other hand, if the person was middle class, for the same vignette, the dissimilarity was medium. The dissimilarity was high if the person was of lower class or vulnerable.

### Intraindividual identity conflict

The intraindividual conflict between gender, occupation and SES was observed by adapting the scale used by Gibson et al. [21]. Gibson’s scale includes information about conflict of group goals, practices conflict, and affiliation conflict. The original scale was translated into Spanish, back translated into English, and then presented to the original author, who approved the definitive version. Afterward, a fourth factor concerning conflict of values was added to the original scale following the guidelines of Ashforth and Schinoff [53] and Ashforth et al. [9].

Then, based on goals, practices, affiliation, and values, four subscales were constructed containing three items that referred to the conflict between gender and SES, SES and occupation, and gender and occupation. The scale included twelve items, using a five-level Likert scale, in which five represented “strongly agree” and one “strongly disagreed.” The total score for identity conflict was obtained from the mean of the responses. The higher the scores, the higher the levels of conflict.

### SES

The socioeconomic classification methodology developed by the AIM [47] was used to identify the SES. This instrument performs socioeconomic classification at the household level in the Chilean economy. It combines economic factors (income) with social factors (education and occupation) associated with status (For example, “What is the highest educational level the head of the household attained (last year)?” “Including yourself, how many people are currently living in your household?” “Please think about your household’s total income in an average month... What is your household’s total monthly income?”).

Based on the responses given by participants, a classification table established by the same methodology was used to assign a socioeconomic group. The SES can take seven levels, including 1 (poor people); 2 (vulnerable people); 3 (lower middle class); 4 (typical middle class); 5 (emerging middle class); 6 (affluent middle class); and 7 (high class). For the purpose of this study, three categories were established: high SES (groups 6 and 7), middle SES (groups 4 and 5), and lower SES (groups 1, 2, and 3)

### Gender

Gender was observed as the self-perceived identity of each individual regarding their gender through the question: “How do you identify your gender?” The question provided the options to identify as female, male, or non-binary.

### Demographic characteristics and control variables

Regarding demographic characteristics and identity, information was collected on age, nationality, experience, education, occupation, and tenure.

To ensure effectiveness in hypothesis testing, the Mor Barak inclusion perception scale [54] was used for control purposes. Inclusion is understood as an individual’s perception of being part of the organizational system, both formal and informal processes, where decisions are made, and information is exchanged and moderates the effects of diversity [54,55]. Considering that people may be developing different processes of inclusion in their work, it was necessary to control them to observe in more detail the effect of diversity.

The English version scale was adapted to Spanish through translation and back-translation [56]. The scale comprised Likert-type indicators of one to five to evaluate the level according to the statements exposed. One equated to “strongly disagree” and five to “strongly agree.” From the original scale, only the dimensions of inclusion within the workgroup and inclusion by the supervisor were utilized. The data collection took place during the COVID-19 pandemic, a period when face-to-face work was suspended in many parts of the world. Consequently, the dimension related to participation in informal spaces was excluded, as it included questions about social interactions that were not feasible during quarantine conditions.

### Data-collection procedure

Contact with the union’s leadership was established via email (11.2.2021), and the research objectives were presented during two virtual meetings. Additionally, to encourage worker participation in the study, we pledged to provide access to aggregated information at the conclusion of the research.

The study information and the online questionnaire, presented in Spanish, were distributed to the sample group through the union’s email. The researchers were responsible for crafting the message’s content and wording, ensuring that the trade union leadership did not influence the message’s content or meaning. The questionnaire format was customized based on the self-reported information about gender, occupation, and SES. The survey remained open for fifteen days, conducted during the first half of January 2022. Responding to the questionnaire typically required about fifteen minutes.

Participants were briefed on the study’s objectives, its various stages, data handling, as well as the principles of anonymity and confidentiality. They were requested to provide a written, online recorded informed consent before completing the survey. To incentivize participation, books related to childhood were offered to the participants.

Before data collection, official written ethical approval was obtained from the Research Ethics Committee of the Universidad de Santiago de Chile (Approval Number: 222/2021) on June 18, 2021.

## Results

### Intraindividual identity conflict scale validation

After applying the questionnaire, reliability tests were performed using Cronbach’s Alpha and construct validity through confirmatory factor analysis. For the reliability test, Cronbach’s Alpha for the scale was 0.9. For the construct validity, the proposed factor analysis model presents good fit rates (RMSEA 0.04; CFI 0.98; TLI 0.98; SRMR 0.04). Regarding factor loads, the estimated parameters are detailed in Table 2.

**Table 2:** CFA results for the intraindividual identity conflict scale.

Dimensions and factors	Estimated parameters
*Value Identity Conflict*	
The values of childcare workers do not match those they (their gender) consider important in life.	0.82^**^
The values of childcare workers do not coincide with those that they (their SES) consider important in life.	1.06^**^
The values of (their gender) do not match those that people of (their SES) consider important in life.	0.83^**^
*Practice Identity Conflict*	
Childcare workers solve problems differently than (their gender).	0.78^**^
Childcare workers solve problems differently than people (of their SES).	0.93^**^
(Their gender) solve problems differently than people (of their SES).	0.81^**^
*Goals Identity Conflict*	
Childcare workers and (their gender) want different things in life.	0.80^**^
Childcare workers and people (of their SES) want different things in life.	0.90^**^
(Their gender) and people (of their SES) want different things in life.	0.89^**^
*Belonging Identity Conflict*	
To be a childcare worker and (their gender) is contradictory.	0.57^**^
To be a childcare worker and belonging to (their SES) is contradictory.	0.85^**^
Being (their gender) and belonging to (their SES) is contradictory.	0.82^**^

* p < 0.05;

** p < 0.01.

### Correlations analysis

A Pearson correlation analysis was conducted to examine the relationships. The results, presented in Table 3, indicate a positive correlation between SES dissimilarity and identity conflict. Additionally, a negative correlation was observed between SES and SES dissimilarity, as well as between SES and identity conflict.

**Table 3:** Bivariate correlations of the variables under study.

1. SES	4.43	1.22	1			
**2. Inclusion**	3.53	0.88	0.17^*^	1		
**3. Identity conflict**	2.32	0.70	‒0.23^**^	‒0.13	1	
**4. Gender dissimilarity**	1.63	0.65	‒0.06	0.03	0.00	1
**5. SES dissimilarity**	2.23	0.91	‒0.37^**^	‒0.08	0.18^*^	‒0.02

* p < 0.05;

** p < 0.01.

### Dissimilarity and intraindividual identity conflict

The study explored the association between gender and SES dissimilarity in relation to intraindividual identity conflict, examining conflict across dimensions of goals, values, practices, and group affiliation. A statistical model was constructed using Structural Equation Modelling (SEM), with identity conflict as the dependent variable and gender as well as SES dissimilarity as independent variables. The model exhibited strong fit indices, indicating its robustness (RMSEA 0.06, 90% CI [0.04, 0.08]; CFI 0.97; TLI 0.96; SRMR 0.04).

A direct and statistically significant relationship between socioeconomic dissimilarity and intraindividual identity conflict was observed. SES dissimilarity was related to values conflict (*β*=0.19, IC 95% [0.04; 0.34]; p <  0.01; R2 = 0.04) and affiliation conflict (*β=*0.15, IC 95% [- 0.01, 0.3]; p <  0.05; R2 = 0.02) that is, being different in a working group regarding SES generates intraindividual conflict between one’s gender, SES, and occupation identities. Conversely, the gender dissimilarity effect on intraindividual identity conflict was not statistically significant. The details of this information are shown in Table 4.

**Table 4:** SEM results of tested models.

	Model 1	Model 2	Model 3
**Gender Dissimilarity → values conflict**	‒0.73		
**Gender Dissimilarity → goals conflict**	0.44		
**Gender Dissimilarity → practices conflict**	0.11		
**Gender Dissimilarity → affiliation conflict**	0.02		
**SES Dissimilarity → values conflict**	0.19^**^	0.17^*^	0.66^*^
**SES Dissimilarity → goals conflict**	0.1	0.60	0.64^*^
**SES Dissimilarity → practices conflict**	0.15	0.71	0.55
**SES Dissimilarity → affiliation conflict**	0.15^*^	0.76	0.17
**SES Dissimilarity X SES → goals conflict**			‒0.60^*^

* p < 0.05;

** p < 0.01.

To better understand this relationship, a second model wherein only SES dissimilarity was measured while controlling demographic factors such as gender, SES, age, and inclusion. Again, the model presented a good fit (RMSEA 0.04, 90% CI [0.03, 0.06]; CFI 0. 97; TLI 0.96; SRMR 0.05), and the relationship between dissimilarity of SES and identity conflict was retained through values (*β=*0.17, IC 95% [0.01, 0.33]; p <  0.05; R2 = 0.1, p < 0.05). Similarly, a direct effect of SES was observed on practices conflict (β =  -0.19, IC 95% [-0.35, - 0.03]; p <  0.05; R2 =  0.17, p < 0.01) and affiliation conflict (β =  -0.21, IC 95% [-0.37, -0.05]; p <  0.01; R2 =  0.19, p < 0.01).

The model also established the direct effect of inclusion on identity conflict. Therefore, the team inclusion harmed identity conflict through values conflict (*β=*-0.20. IC 95% [-0.4,0.00]; p <  0.05; R2 = 0.1, p < 0.05), practices conflict (*β=*-0.39, IC 95% [-0.57,-0.20]; p <  0.01; R2 = 0.17, p < 0.01), goals conflict (*β=*-0.31, IC 95% [-0.40.0.00]; p <  0.001; R2 = 0.12, p < 0.05), and affiliation conflict (*β=*-0.38, IC 95% [-0.57,-0.12]; p <  0.000; R2 = 0.19, p < 0.01). Also, a direct effect of supervisor inclusion on identity conflict was observed through goals conflict (*β=*0.25, IC 95% [0.05; -0.45]; p <  0.01; R2 = 0.12, p < 0.05) and affiliation conflict (*β=*0.27, IC 95% [0.08,0.46]; p <  0.01; R2 = 0.19, p < 0.01).

The results demonstrate that being different from a group concerning SES directly correlates with intraindividual identity conflict. Such findings correspond with H2: SES diversity, understood as dissimilarity, has a direct, positive, and significant relationship with intraindividual identity conflict. At the same time, the effect of gender dissimilarity on identity conflict was not supported.

### Moderation effect on dissimilarity and intraindividual identity conflict

In the case of the moderating role of the SES in the relationship between SES dissimilarity and identity conflict, we ran a third model, a regression adding SES while controlling for gender, age, and inclusion. In this case, the model showed a good fit (RMSEA 0.04, 90% CI [0.02, 0.05]; CFI 0.97; TLI 0.96; SRMR 0.05) and confirmed the effect of dissimilarity on the identity values conflict (*β=*0.66, IC 95% [0.06, 1,3]; p <  0.05; R2 = 0.12, p < 0.05) and goal conflict (*β=*0.64, IC 95% [0.05, 1,2]; p <  0.05; R2 = 0.15, p < 0.01). The results also showed that the relationship between SES dissimilarity and identity conflict depends on the individual’s SES. Specifically, SES negatively moderates the relationship between dissimilarity and goals conflict (*β=*-0.6, IC 95% [‒1,2, 0.00]; p < 0.05; R2 = 0.15, p < 0.01). The results showed a negative direct effect of inclusion on identity conflict and a positive effect through supervisor inclusion.

**Fig 2:**
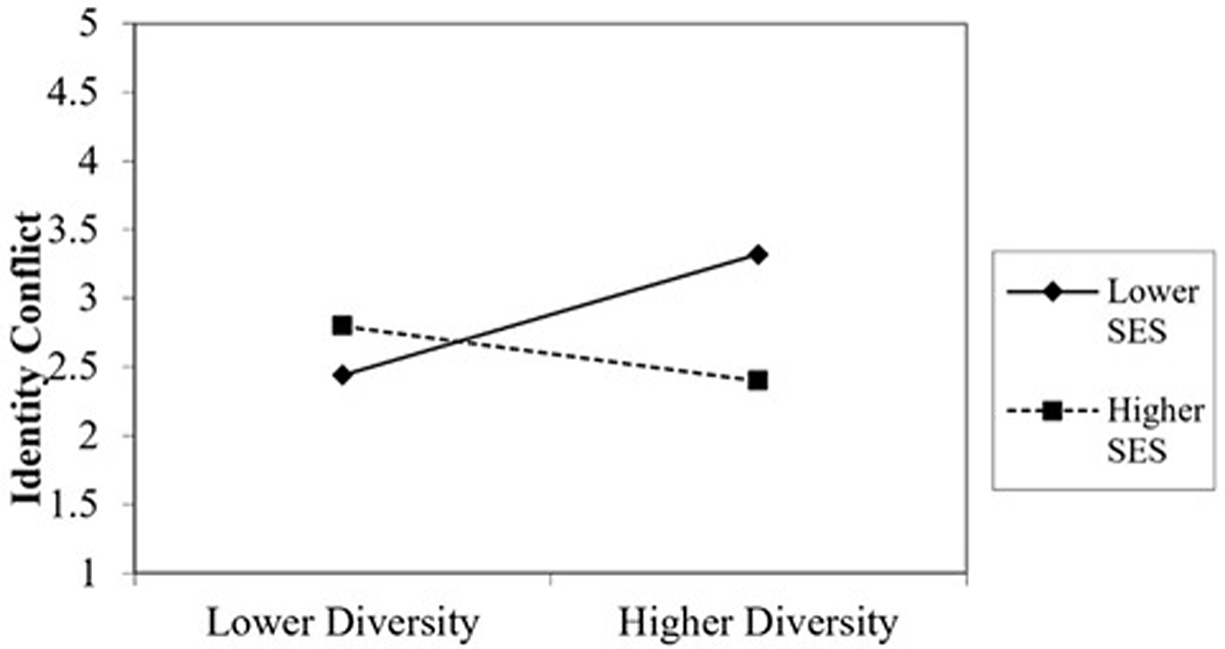
Moderating effect of SES.

In sum, the effect of diversity on intraindividual identity conflict will be more pronounced in people with lower SES than higher SES. Consequently, the study provides evidence to support H4: The effect of socioeconomic diversity on intraindividual identity conflict has a negative relationship with SES (Fig 2).

**Fig 3:**
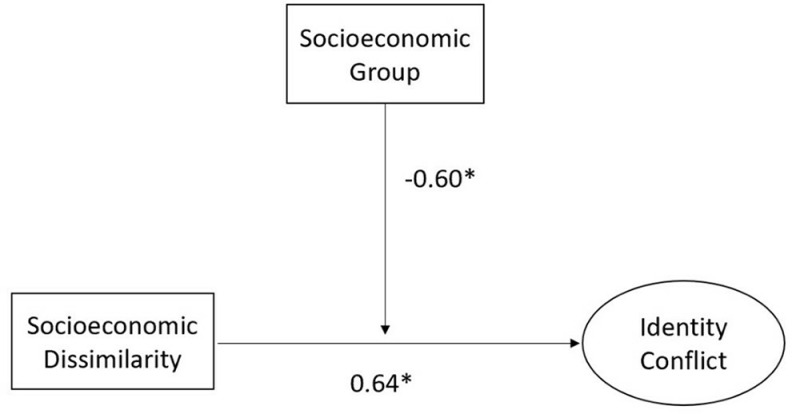
Modified hypothetical path model.

It can be concluded that being different regarding SES is related to intraindividual identity conflict between a person’s gender, occupation, and SES, and this relation depends on the person’s SES (Fig 3). At the same time, it was not possible to observe the effect of gender diversity on intraindividual identity conflict in a feminized occupation. Finally, our results confirm the relevance of SES diversity and the idea that diversity outcomes depend on the status of the observed identity.

## Discussion

This study reveals the effect of SES dissimilarity on intraindividual identity conflict between a person’s occupation, gender, and SES in a female dominated sector. Likewise, the moderating role of the SES between diversity and intraindividual identity conflict was confirmed. Indeed, the results show that being different from the working group regarding one’s SES implies that identities can come into conflict.

These results are relevant for several reasons. Firstly, this is one of the first studies to observe the results of SES diversity among working groups. In so doing, this study studied a category of identity that, despite its relevance [42], is not commonly included in diversity studies [57].

Indeed, SES differences have been mainly integrated within studies on discrimination in recruitment and selection processes [58], but studies looking at socioeconomic diversity are limited. The reasons for this omission cannot be confirmed, but according to Jonsen et al. [59], it seems that it is not a relevant issue in some cultures. Based on research elsewhere, however, such a conclusion seems tenuous, as the effect of SES on distinct aspects of management worldwide has been recognized [60].

In parallel, the omission of SES diversity within research can be linked to the origins of diversity studies related to the civil rights movement in the United States [5]. Moreover, these early investigations played a relevant role in diversity studies [61], focusing mainly on discrimination processes revolving around entering and remaining in organizations [62]. Thus, the study of traditionally disadvantaged groups—such as people of color, women, and, in this case, people of low SES or working class—has been carried out with a focus on their integration into the workplace and not necessarily on their development and well-being.

This study shows that, in addition to SES effects on career development [63], SES diversity affects individuals through intraindividual identity conflict. Therefore, it is relevant, if not necessary, to integrate SES or class within analyses of demographic diversity. Research focusing on diversity SES is needed because it is a category related to intersectional identities. Indeed, SES would represent the status and power relations embedded in identities like gender, race, or nationality [64]. Considering SES diversity is also necessary because, in contexts of low social mobility, such as Chile, Brazil, and other developing countries [65,66], it is a source of intergroup social conflicts, affecting group cohesion and performance. Thus, this study calls attention to SES and social class because it is an important component of barriers to entry and other processes, such as inclusion within the workgroup and participation in decision-making.

In addition to the importance of SES concerning diversity, the research revealed a relationship between diversity and intraindividual identity conflict. Therefore, it is possible to say that diversity generates conflict between identities within groups [6,67] and within person. From the social identity theory perspective, individuals would seek to be part of groups of identities that they value to strengthen their self-concept and self-esteem in the categorization and self-categorization process. However, this search can also lead to intraindividual conflicts since this search for identities -which are multiple- will generate tensions between them.

Intraindividual identity conflict has been proven to be an important factor in the workplace, as it is related to detrimental outcomes on performance and motivation [20], well-being and performance [18], and stress and life satisfaction [38], among others. In light of such findings, this study’s results allow us to project that the harmful results of diversity would not only correspond to group identity conflict but also to the individual conflicts that people develop by belonging to and identifying with different groups [21].

These results contribute to research about diversity, conflict, and status. As other studies have shown [36,68,69], difference manifests in different effects for different people; in particular, the difference has more pronounced consequences for those of lower status.

The study also observed the role of inclusion—in this case, within the group. A greater perception of inclusion is related to a lower intraindividual identity conflict generated by dissimilarity with the group. This result adds to the studies that have analyzed the effect of a climate of inclusion on group conflict caused by diversity [3,36,54,67], confirming the idea that being different—regarding gender, race, and, as evidenced by the present study, SES—brings about conflict for people and groups and that it is, therefore, necessary to create and encourage inclusive environments.

Notably, this is one of the few studies to study workplace diversity using experimental techniques. Furthermore, using vignettes permits us to assess the effect of diversity while controlling the effect of other diversities not considered in observational studies. Therefore, this study contributes to the theoretical development of the construct of diversity and its consequences using a more robust methodological design that ensures greater reproducibility.

Furthermore, this study stands out as one of the limited investigations examining diversity within a developing nation’s framework, determined by the country’s historical, socioeconomic, and political development. Presenting findings from Chile, a representative of the global south, underscores that diversity is a universal phenomenon. While it may manifest uniquely in different local contexts, addressing diversity on a global scale is imperative to mitigate its adverse effects and enhance its potential benefits.

It is important to note that no gender diversity effects were observed in a feminized organization. While this limitation restricts the scope of our analysis of the phenomenon under study, our research once again reaffirms the necessity of examining the impact of gender diversity in feminized industries. Within this sector, men also hold leadership positions and employ frontline roles as steppingstones for advancing into manager careers [70]. Consequently, it is reasonable to anticipate that gender diversity will yield distinct outcomes. Further research in this area is warranted.

Lastly, acknowledging this situation from a feminist perspective entails not simplifying the issue by merely placing more women in leadership positions or increasing the hiring of men. In light of the growing discourse on gender parity, we believe that coercing women into positions of power may give rise to new forms of discrimination, especially as more marginalized women enter the caregiving industry. From an intersectional viewpoint, gender diversity is not solely a gender concern but also one of class and power dynamics [71].

### Limitations and future research

Although this study enables the analysis of hypotheses, it is crucial to interpret the results cautiously, as the identity conflict primarily focused on specific identities. Moreover, the sample size and the low male representation limited hypothesis testing related to gender diversity. Therefore, future comparative studies on gender diversity in female-dominated occupations should be conducted with larger and more representative samples. Moreover, while the context of women’s work is crucial in the existing body of research, it would be valuable for future studies to include a counterfactual group to enhance the robustness of the analyses.

It is also important to consider that the study population exhibits specific characteristics regarding their goals and values, particularly the documented public service motivation among these workers [72,73]. This may result in a unique dynamic concerning identity conflicts related to goals and values. Therefore, incorporating other occupations into future research appears crucial to gaining a deeper understanding of this phenomenon.

Additionally, the results concerning SES diversity’s effect on identity conflict demonstrate a consistent impact only on goal conflict, and it does not appear as a solid effect across multiple identities in conflict. Considering the novelty of the current findings presented in this study, future studies should deepen the investigation of the specificity of identity conflict and explore the conditions under which certain types of intraindividual conflicts emerge.

On the other hand, the ecological validity of the study is limited. Although the vignette design allows for control, it does not guarantee that variables behave similarly in a natural context. Future studies should employ longitudinal models to validate or refute some of the findings presented here to address this limitation. Furthermore, as this was an experimental study conducted online, it is essential to examine the behavior of some variables in a laboratory setting. Ideally, models based on simulations or random groups in the laboratory could be utilized to deepen the insights derived from the reported findings.

Simultaneously, new research should endeavor to acknowledge a broader spectrum of identities. This is imperative because in our study, identities were presented as binary. In this regard, future investigations need to challenge the prevailing view of gender rooted in binary cisnormativity [74]. Lastly, it is important to acknowledge that the sample used in this study may not comprehensively represent other populations not included in the current research.

## Conclusions

Differences continue to generate conflict if diversity and inclusion in the workplace are not established and developed. Being part of more diverse groups entails group conflicts, and as demonstrated by this study, being different from the group often results in intraindividual identity conflicts. Thus, the challenge remains to advance the development of inclusive climates and promote inclusive leadership that recognizes differences and offers equal opportunities and equitable treatment.

This study’s contributions concerning the effects of SES diversity highlight the significance of considering class diversity as a relevant determinant of organizational outcomes. Despite the strength of other identities, in contemporary societies, socioeconomic dimensions have both a direct and indirect explanatory weight regarding questions of individuality and group identity.

Lastly, our study emphasizes the need to move beyond diversity management approaches that reproduce the social order in order to promote inclusion from the perspective of optimal distinctiveness [75], where individuals are recognized for their uniqueness and treated equally.
